# Supplementary material for: Serum Antibody Activity against Poly-N-Acetyl Glucosamine (PNAG), but Not PNAG Vaccination Status, Is Associated with Protecting Newborn Foals against Intrabronchial Infection with Rhodococcus equi
Source: Microbiol Spectr. 2021 Jul 28;9(1):10.1128/spectrum.00638-21. doi: 10.1128/spectrum.00638-21 (PMC8552712; doi:10.1128/spectrum.00638-21)
Supplement: SUPPLEMENTAL FILE 1 — Supplemental material. Download SPECTRUM00638-21_Supp_1_seq10.pdf, PDF file, 0.3 MB. [file spectrum00638-21_supp_1_seq10.pdf]

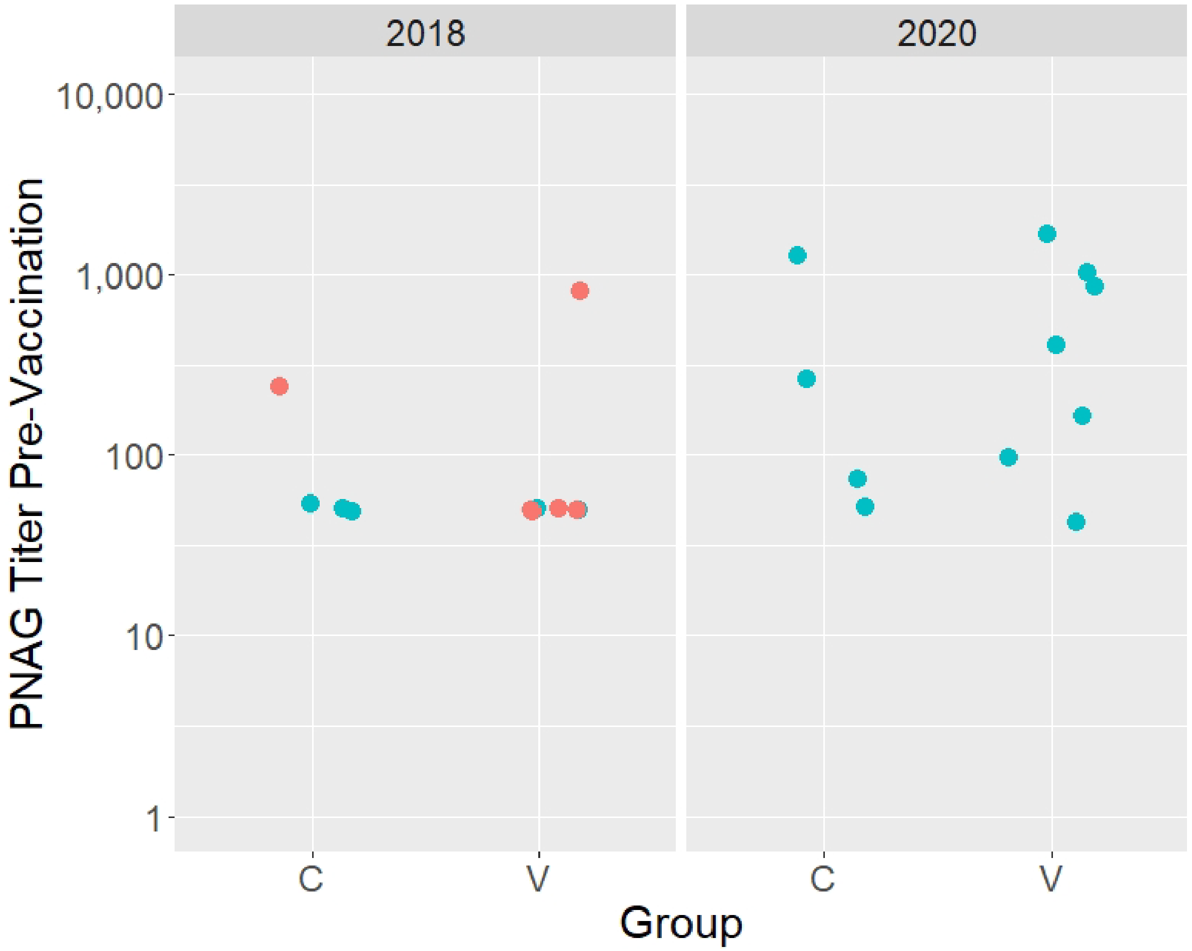

Supplemental Figure 1. Antibody activities against PNAG in sera from mares obtained before vaccination, faceted by year (2018 or 2020). Group represents vaccination status (C= Control; V = Vaccinated against PNAG). Teal circles are foals that developed pneumonia and coral circles are foals that remained healthy.

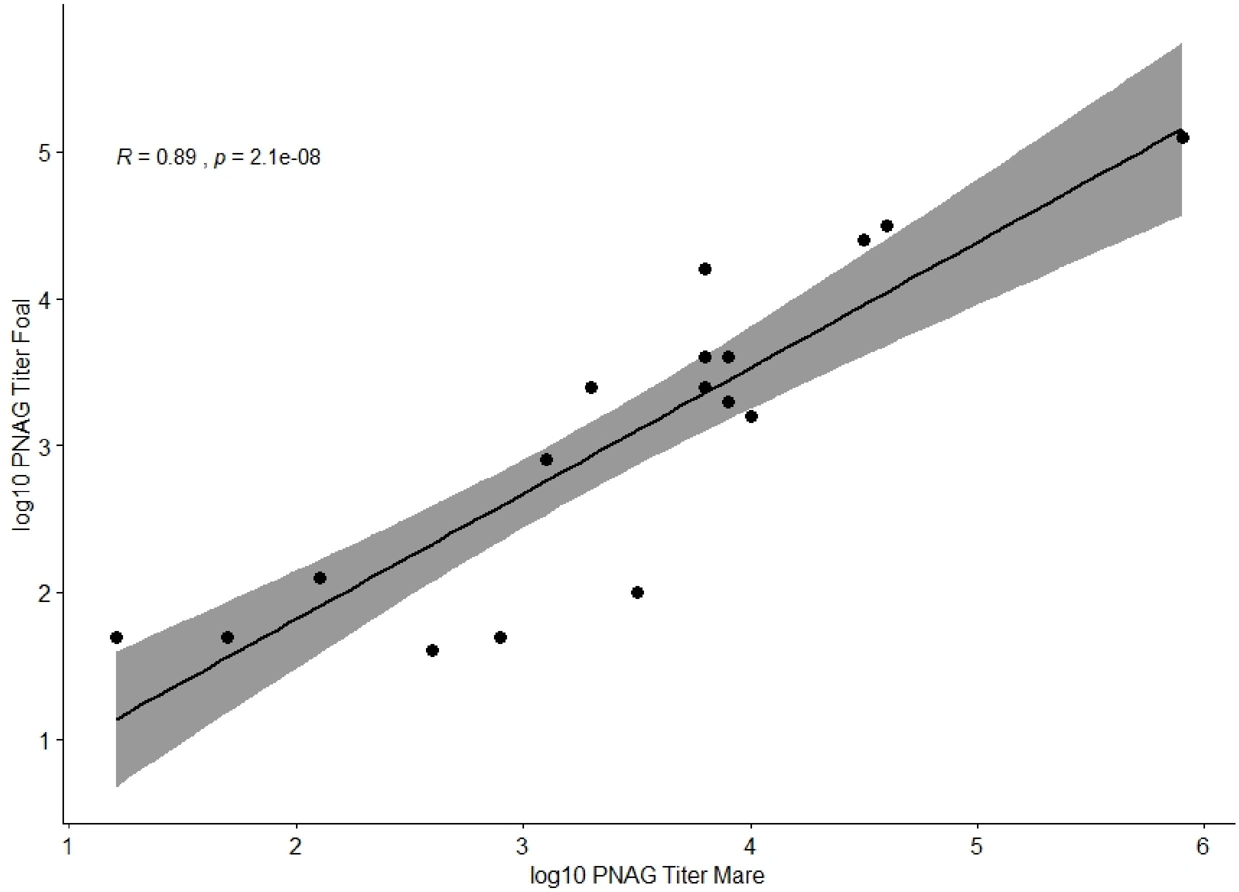

Supplemental Figure 2. Correlation between antibody activities to PNAG in sera from mares and their foals when foals were approximately 24 hours old.

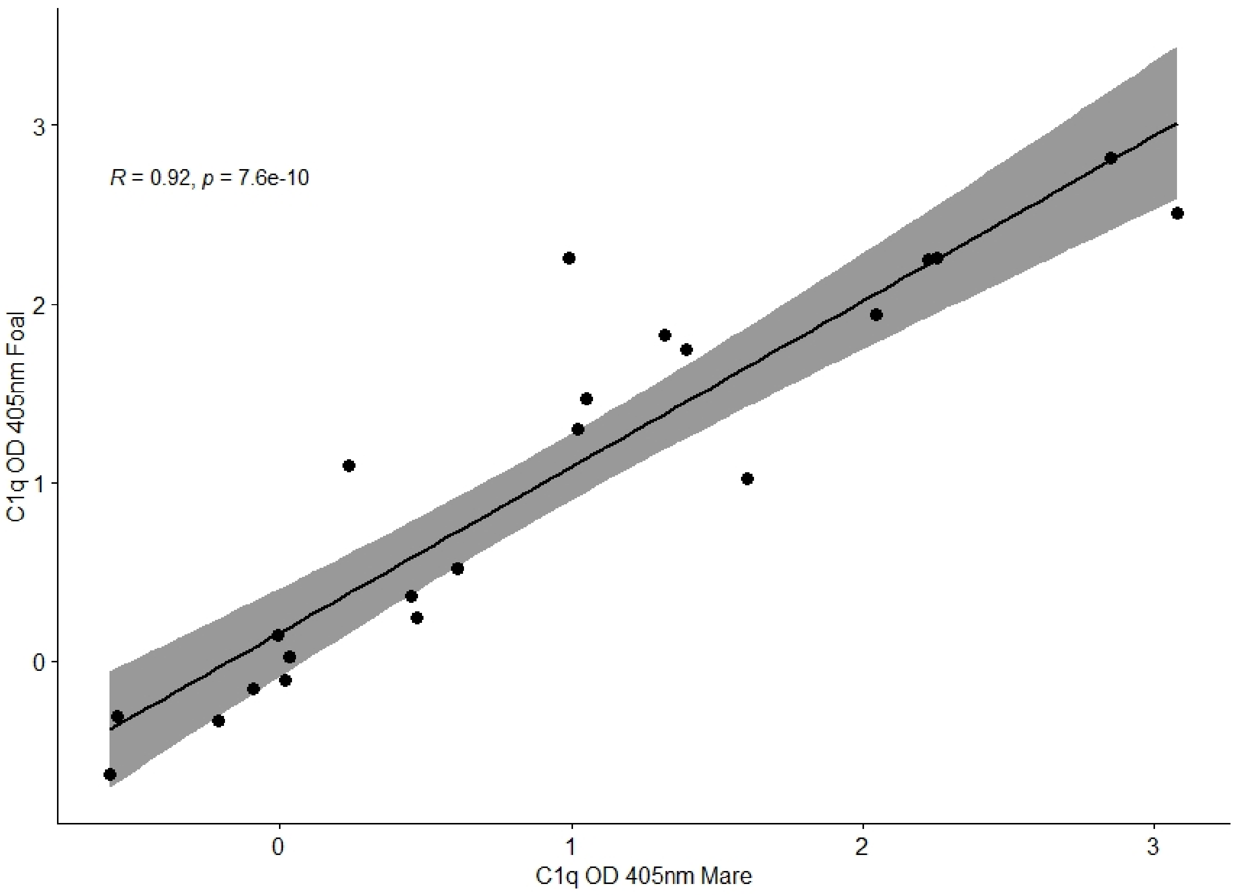

Supplemental Figure 3. Correlation between C’1q deposition activity in sera from mares and their foals when foals were approximately 24 hours old.
